# Identification of lung adenocarcinoma subtypes and a prognostic signature based on activity changes of the hallmark and immunologic gene sets

**DOI:** 10.1016/j.heliyon.2024.e28090

**Published:** 2024-03-24

**Authors:** Shun-Kai Zhou, De-Hua Zeng, Mei-Qing Zhang, Meng-Meng Chen, Ya-Ming Liu, Qi-Qiang Chen, Zhen-Ya Lin, Sheng-Sheng Yang, Zhi-Chao Fu, Duo-Huang Lian, Wen-Min Ying

**Affiliations:** a Department of Thoracic and Cardiac Surgery, The 900th Hospital of the Joint Logistics Support Force of the People's Liberation Army, Fuzhou, Fujian Province, 350000, China; b Department of Pathology, 900th Hospital of the Joint Logistics Support Force of the Chinese People's Liberation Army, Fuzhou, Fujian Province, 350000, China; c Department of Anesthesiology, The 900th Hospital of the Joint Logistics Support Force of the Chinese People's Liberation Army, Fuzhou, Fujian Province, 350000, China; d Department of Radiotherapy, The 900th Hospital of the Joint Logistics Support Force of the Chinese People's Liberation Army, Fuzhou, Fujian Province, 350000, China; e Department of Radiotherapy, Fuding Hospital, Fuding City, Fujian Province, 355200, China

**Keywords:** Lung adenocarcinoma, Subtype, Hallmark and immunologic gene sets, Prognostic signature, Immune microenvironment, Prognosis

## Abstract

**Background:**

Lung adenocarcinoma (LUAD) has a complex tumor heterogeneity. Our research attempts to clearness LUAD subtypes and build a reliable prognostic signature according to the activity changes of the hallmark and immunologic gene sets.

**Methods:**

According to The Cancer Genome Atlas (TCGA) - LUAD dataset, changes in marker and immune gene activity were analyzed, followed by identification of prognosis-related differential gene sets (DGSs) and their related LUAD subtypes. Survival analysis, correlation with clinical characteristics, and immune microenvironment assessment for subtypes were performed. Moreover, the differentially expressed genes (DEGs) between different subtypes were identified, followed by the construction of a prognostic risk score (RS) model and nomogram model. The tumor mutation burden (TMB) and tumor immune dysfunction and exclusion (TIDE) of different risk groups were compared.

**Results:**

Two LUAD subtypes were determined according to the activity changes of the hallmark and immunologic gene sets. Cluster 2 had worse prognosis, more advanced tumor and clinical stages than cluster 1. Moreover, a prognostic RS signature was established using two LUAD subtype-related DEGs, which could stratify patients at different risk levels. Nomogram model incorporated RS and clinical stage exerted good prognostic performance in LUAD patients. A shorter survival time and higher TMB were observed in the high-risk patients.

**Conclusions:**

Our findings revealed that our constructed prognostic signature could exactly predict the survival status of LUAD cases, which was helpful in predicting the prognosis and guiding personalized therapeutic strategies for LUAD.

## Highlights

• Two molecular subtypes of LUAD were identified.
• Cluster 2 showed worse prognosis and higher immune infiltration than cluster 1.
• Use two subtype related DGS to set up a prognostic risk scoring model.
• Compared to the low-risk group, the high-risk group has poorer survival rates and higher TMB.
• Nomogram structured according to the risk score and clinicopathological data can predict survival.

## 1 Introduction

Lung cancer is a malignant tumor that threatens the death of all humans. According to a published report in 2020, the global lung cancer burden has increased, with 2.2 million (11.44%) new cases and 1.8 million (18%) deaths. Moreover, about 75% of lung cancer cases are diagnosed as advanced, leading to bad overall survival (OS) (6% and 37%, respectively) [1,2]. Non-small cell lung cancer (NSCLC) is the main lung cancer type, of which lung adenocarcinoma (LUAD) is the most common subtype having complex tumor heterogeneity in clinical, molecular, cellular and tumor behavioral level [3,4]. Intratumor heterogeneity of LUAD remains a confusing factor responsible for the inaccurate diagnosis, treatment and prognostic assessment [5]. For patients with LUAD, individualized treatment strategies will help to achieve good clinical outcomes [6]. Therefore, identification of LUAD subtypes and a reliable prognostic signature will improve patient's clinical outcomes through personalized treatment and accurate prognosis assessment.

Cancer is a intricate disease involving intricate regulations between the cancer and the immune system [7]. The immune system is indispensable in the occurrence and development of cancer, and evading immune destruction is considered a cancer hallmark. The breakthrough in immunotherapy has greatly benefited some patients with lung cancer [8,9]. Recently, many research studies have focused on discovering immune-related prognostic gene sets in various solid tumors that are related to immunotherapy response and cancer prognosis [10]. For instance, a prognostic signature constructed from 10 immune-related genes shows great performance in predicting disease prognosis and can improve the immunotherapy management in LUAD [11]. Song et al. also constructed a prognostic signature comprising 30 immune-related genes, which had a high prognostic value in patients with LUAD and promoted individualized treatment [12]. In addition, cancer complexity is represented by numerous cancer hallmarks that allow cancer cells to proliferate and metastasize. Cancer hallmark genes are responsible for crucial phenotypic features associated with the malignant development of cancers, and signatures established by cancer hallmark genes are important in various cancer types [13,14]. A hallmark-based six-gene levels can reflect the genetic characteristics of colorectal cancer cells and can be used to evaluate the recurrence risk of colorectal cancer [15]. Moreover, a robust six-gene prognostic signature that is remarkably associated with cancer hallmarks is reliable for predicting OS in NSCLC [16]. These data suggest the potential of immunologic and hallmark gene sets as promising biomarkers for cancer prognosis. However, there is still limited research on crucial hallmark and immunologic gene sets related to LUAD prognosis.

**Fig. 1:**
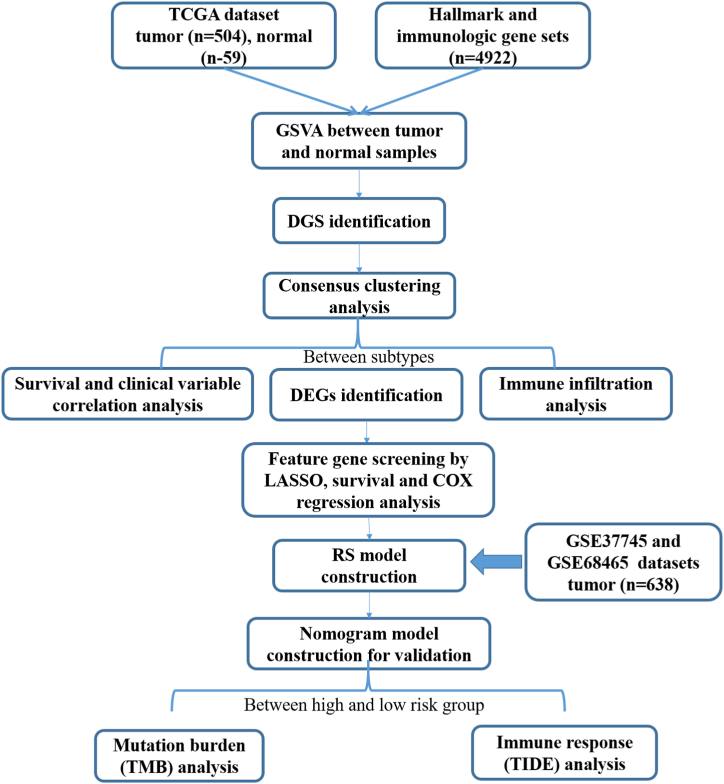
Flow chart of bioinformatic analysis.

Changes in the activities of the hallmark and immunologic gene sets may have a crucial impact on lung cancer prognosis by affecting the colonization of circulating tumor cells in blood and their proliferation after therapeutic interventions, such as pulmonary celiectomy. Nevertheless, few studies have determined LUAD subtypes or prognostic gene sets according to changes in the activity of marker and immunologic gene sets. Herein, we analyzed the changes in the activity of marker and immunologic gene sets on basis of the Cancer Genome Atlas (TCGA)-LUAD dataset, followed by identification of prognosis-related differential gene sets (DGSs) and their related LUAD subtypes. Survival analysis, correlation with clinical characteristics, and immune microenvironment assessment for the subtypes were performed. Moreover, the DEGs (differentially expressed genes) between different subtypes were confirmed, next, a prognostic risk score (RS) model and validated nomogram model construction. Additionally, two Gene Expression Omnibus (GEO) datasets (GSE37745 and GSE68465) were attempts to validate the predictive accuracy of the prognostic model. Furthermore, the tumor mutation burden (TMB) levels were compared between different risk groups. Our bioinformatics analysis flowchart is depicted in Fig. 1. Our findings provide more in-depth insight to accurately assess the prognosis and improve the therapeutic interventions for LUAD.

## 2 Materials and methods

### 2.1 Data acquisition

The gene expression RNA-sequencing (RNA-seq) and clinicopathological parameters of LUAD were downloaded from TCGA database. This dataset included 504 cancerous and 59 normal samples.

Gene expression pattern of lung cancer with prognostic information, namely GSE37745 and GSE68465, were obtained from the GEO database. By de-batch integration, 638 lung cancer samples from the two datasets were used for subsequent analyses.

Furthermore, 4922 hallmark and immunologic gene sets were obtained from the Molecular Signatures Database (MSigDB) [17] using Gene set enrichment analysis (GSEA) (https://www.gsea-msigdb.org/gsea/msigdb/index.jsp).

### 2.2 Gene set variation analysis (GSVA)

Applying GSVA to observe changes in gene pathway activity under specific biological conditions by estimating the relative enrichment of a group of genes in a sample population [18]. Based on the sample information in TCGA-LUAD dataset, the enrichment score (ES) of 4922 hallmark and immunologic gene sets were analyzed using GSVA [18] to uncover changes in the activities of the marker and immunologic gene combinations in LUAD relative to normal condition.

### 2.3 Identification of DGSs

Based on the ESs of gene sets in TCGA-LUAD samples, the DEGs between cancer and non-cancer specimen were analyzed applying the *t*-test in R4.1.2, with a cut-off value of *P* < 0.01. Moreover, prognosis-related gene sets were screened using univariate Cox regression analysis in the survival package (version 3.2.13) [19] in R 4.1.2, with a threshold value of *P* < 0.01. By intersection analysis of DEGs and prognosis-related gene sets, prognosis-related differentially expressed genes (DGSs) were obtained.

### 2.4 Subtype analysis using consensus clustering

According to DGSs, lung cancer samples were clustered using an unsupervised clustering method with the ConsensusClusterPlus package (version 1.58.0) [20] in R4.1.2. Divide the specimens into k [[2], [3], [4], [5], [6], [7], [8], [9]] groups to explore the k value responsible for the stability of the clustering. The best k value was verified via the proportion of ambiguous clustering (PAC).

### 2.5 Survival analyses for subtypes

To determine whether there was different prognosis between subtypes, Kaplan–Meier (KM) survival analyses for patients in different subtypes were conducted via the survival package (version 3.2.13) [19] in R 4.1.2. *P* < 0.05 was set as the cutoff value.

### 2.6 Relationship between subtypes and clinicopathological features

According to the clinical data of samples in each subtype, the association between subtypes and different clinicopathological data, including gender, TNM staging, and clinical stage, was assessed using the Chi-square test.

### 2.7 Immune microenvironment assessment for subtypes

Based on the expression levels in each specimen in the TCGA-LUAD dataset, the proportion of 22 immune cell types was estimated via the CIBERSORT algorithm [21]. Compare the differences in immune cells (DICs) between different subtypes. In addition, the estimate package [22] in R4.1.2 was applied to estimate the immune, stromal, ESTIMATE scores and tumor purity of each specimen. The differences in stromal and immune scores between different subtypes were estimated using the Wilcoxon test.

### 2.8 Identification of differentially expressed geness (DEGs) between subtypes

According to tumor subtype information, the DEGs between different subtypes were assessed using the limma package (version 3.50.0) [23] in R4.1.2. The cut-off values were *P* < 0.01 and |log_2_FC (fold change)| > 0.25.

### 2.9 Constitution and appraisement of a prognostic RS model

For subtype-related DEGs, least absolute shrinkage and selector operation (LASSO) regression analysis was used to select feature gene sets using the glmnet package (version 4.1.3) [24] in R4.1.2. Subsequently, according to the ESs of the feature gene sets, they were classified into high- and low-ES groups. Survival analysis was performed to screen the prognosis-related gene sets. Subsequently, perform stepwise Cox regression analysis on gene sets with *P* < 0.01, and a prognostic RS model was established using the following formula:

$$\text{RS}=h_{0}\;\left(t\right)\times \exp\left(\beta_{1}X_{1}+\beta_{2}X_{2}+\ldots +\beta_{n}X_{n}\;\right)$$

where β is the regression coefficient, h_0_ (t) is the benchmark risk function, and h (t, X) is the risk function related to the X covariable at time.

Using this expression, the RSs of samples in TCGA-LUAD training dataset and GEO validation datasets were analyzed. According to the median of RSs, divide all specimen into high or low risk group. Then conduct survival analysis to assess the prognosis between different risk groups.

### 2.10 Nomogram construction

To study the prognostic factors, RSs and clinicopathology factors (age, sex, pathologic M, N, and T, and clinical stage) in TCGA-LUAD dataset were conducted to univariate and multivariate Cox regression analyses. Variables with *P* < 0.01 were selected as independent prognosis factors. Then, a nomogram was conducted via the rms package (version 6.2.0) [25]. Concordance index (C-index) and area under ROC curve (AUC) for 1-, 3-, and 5‐year survival probabilities were analyzed to assess the predictive performance of nomogram model. *Analysis of the mutation status between different risk groups*.

• Using the samples in TCGA-LUAD dataset, the mutation information of every gene in the sample was counted, and the top 20 significantly mutated genes with large mutation numbers were selected. Moreover, the mutation frequencies of these significantly mutated genes were assessed in all LUAD specimen via the maftools package (version 2.8.0) in R4.1.2. Moreover, the tumor mutation burden (TMB) levels of all tumor samples were calculated, and the differences between different risk groups were compared.
• *Difference of immunotherapy response between high and low risk groups*

Based on the mRNA expression matrix, TIDE score (tumour immune dysfunction and exclusion) of every specimen was calculated and the difference of TIDE between high and low risk group was analyzed via stat_compare_means function in ggpubr package (version 0.6.0). Difference with *P* < 0.05 was considered significant.

### 2.11 Statistical analysis

The bioinformatic analyses were achieved via the packages in R 4.1.2. The DGSs between cancer tissues and non-cancer controls were assessed by *t*-test. Differential expression of genes between different subtypes were estimated via limma package Version 3.50.0. For DGSs and DEGs identification, *P* < 0.01 was considered as the cutoff value. Survival analysis was performed based on the KM method in survival package Version 3.2–13 with *P* < 0.05 as the threshold value. Cox regression analysis was executed with the implementation of survival package. Factors with *P* < 0.01 was identified as significantly different.

## 3 Results

### 3.1 Activity changes of gene sets in lung cancer specimens

**Fig. 2:**
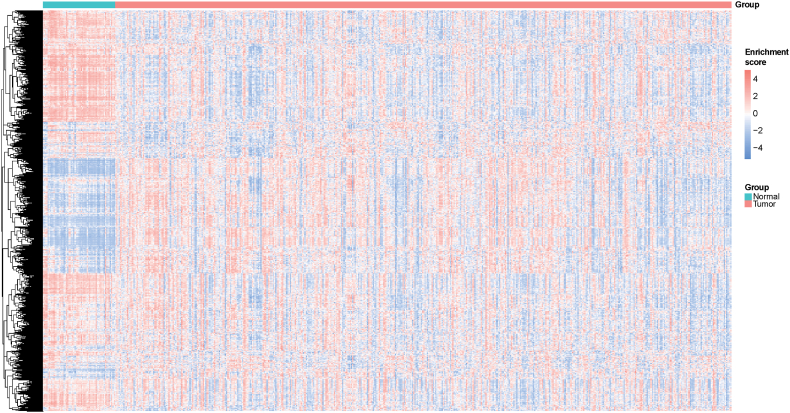
Heatmap of the enrichment scores of 4922 hallmark and immunologic gene sets in tumor and normal samples. Gene set variation analysis (GSVA) was conducted based on The Cancer Genome Atlas (TGCA)-lung adenocarcinoma (LUAD) dataset.

To reveal changes in the activities of the hallmark and immunologic gene sets in lung cancer and normal specimens, GSVA was conducted to assess the enrichment score of 4922 hallmark and immunologic gene sets. The differential enrichment score of hallmark and immunologic gene sets between tumor and non-tumor specimens are shown in Fig. 2.

### 3.2 Identification of prognosis-related DGSs

According to the enrichment score of 4922 gene sets, 4048 DGSs between tumor and non-cancer samples were identified. Moreover, 1054 prognosis-related gene sets were obtained. By further intersection analysis, 955 prognosis-related DGSs were obtained.

Two subtypes were clustered based on prognosis-related DGSs, which had different survival and clinicopathology phenotypes.

**Fig. 3:**
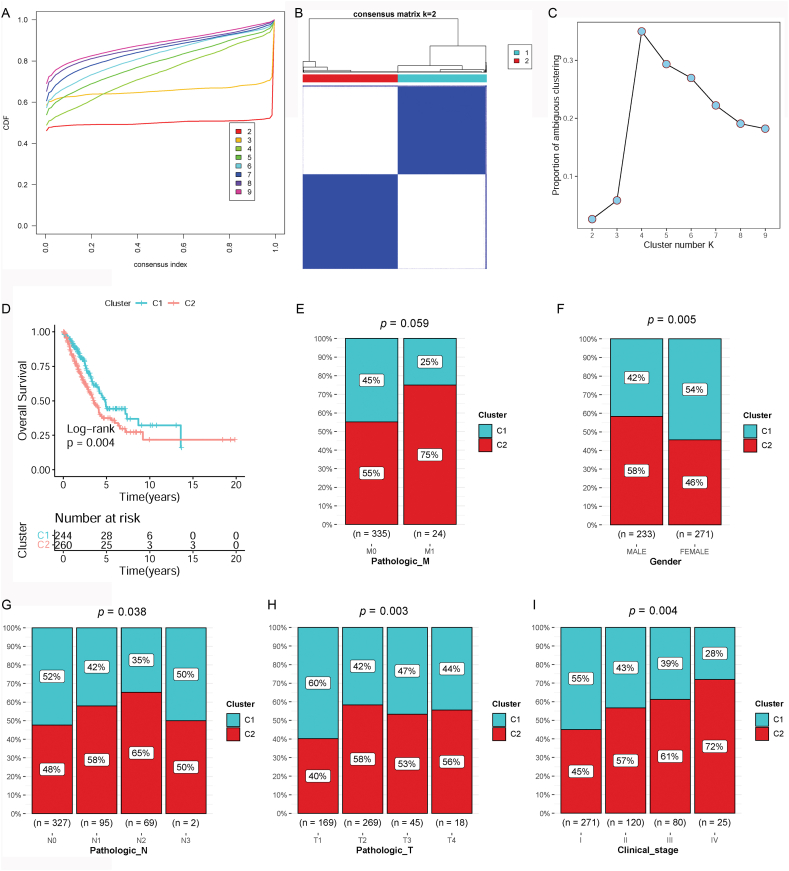
Identification, survival analysis, and clinical correlation of subtypes. A: The cumulative distribution function (CDF) curve of consensus clustering. B: Heatmap of subtype clustering. C: Proportion of ambiguous clustering (PAC) analysis. D: Kaplan–Meier (KM) survival analyses for each subtype. E: Correlation analysis of subtypes and pathologic M stage. F: Correlation analysis of subtypes and gender. G: Correlation analysis of subtypes and pathologic N stage. H: Correlation analysis of subtypes and pathologic T stage. I: Correlation analysis of subtypes and clinical stage.

According to prognosis-related DGSs, lung cancer samples were clustered into two subtypes using unsupervised consensus clustering analysis (Fig. 3A and B). Clusters 1 and 2 contained 244 and 260 lung cancer samples, respectively. Further PAC analysis indicated that the optimal k value was 2 (Fig. 3C).

In the light of the clinicopathology information of specimens clustered in different subtypes, KM survival analysis was conducted to determine if there is a difference in prognosis between the two subtypes. The results showed that the samples from group 2 showed worse prognosis than those from group 1 (p = 0.004, Fig. 3D). Then, conduct relationship analysis between grouping and clinicopathology phenotypes was performed. No pathological differences were found between the two subtypes (Fig. 3E). The clustering of subtypes was significantly related to gender, pathologic N, pathologic T, and clinical stages. Compared with cluster 1, cluster 2 contained more male samples, more samples with N2–N3 stage, more specimens with T2–T4 stage, and more samples with II –IV clinical stage (Fig. 3F–I). These data suggest that the samples of cluster 2 had relatively high degree of malignancy compared to those of cluster 1, which may lead to their bad prognosis.

### 3.3 Difference of immune microenvironment between subtypes

**Fig. 4:**
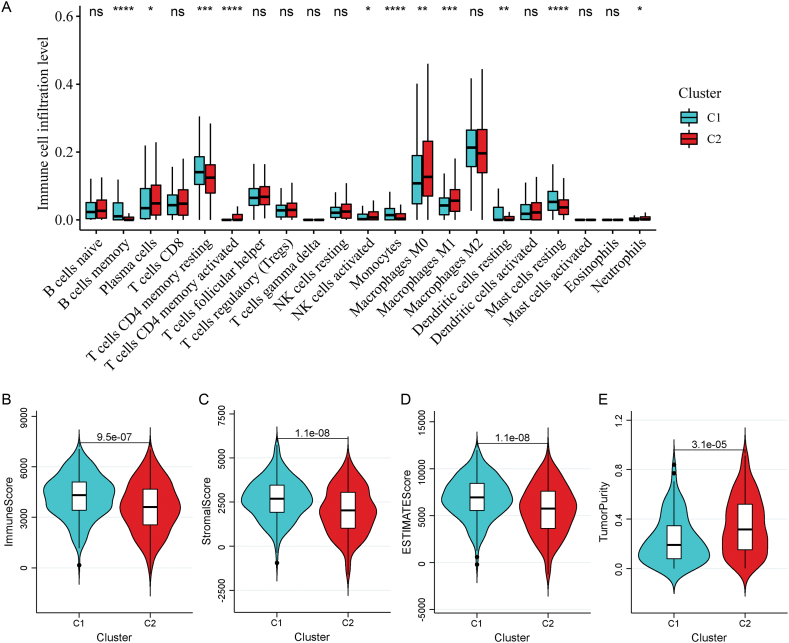
Analysis of the tumor immune microenvironment of two subtypes. A: Comparison of the infiltration levels of 22 types of immune cells between two subtypes. *p < 0.05, **p < 0.01, ***p < 0.001, and ****p < 0.0001. B: Comparison of the immune scores between two subtypes. C: Comparison of the immune scores between two subtypes. D: Comparison of the stromal score between two subtypes. E: Comparison of the ESTIMATE score between two subtypes. F: Comparison of the tumor purity between two subtypes.

To investigate whether there was a significant difference in the tumor immune microenvironment between the two subtypes, the proportion of 22 types of immune cells was quantified on the basis of the expression data in TCGA-LUAD. Eleven DICs were identified between the two subtypes. The abundance of plasma cells, activated memory cluster of differentiation (CD)-4 T cells, activated natural killer (NK) cells, M_0_ macrophages, M_1_ macrophages, resting dendritic cells, and neutrophils was obviously elevated in cluster 2, whereas that of resting memory CD4 T cells, memory B cells, monocytes, and resting mast cells was remarkably increased in cluster 1 (Fig. 4A). Moreover, the stromal, immune, and ESTIMATE scores were significantly lower in cluster 2 than in cluster 1 (Fig. 4B–D), while the tumor purity was dramatically increased in cluster 2 (Fig. 4E).

### 3.4 The prognostic RS model was built by two subtype-related DGSs

**Fig. 5:**
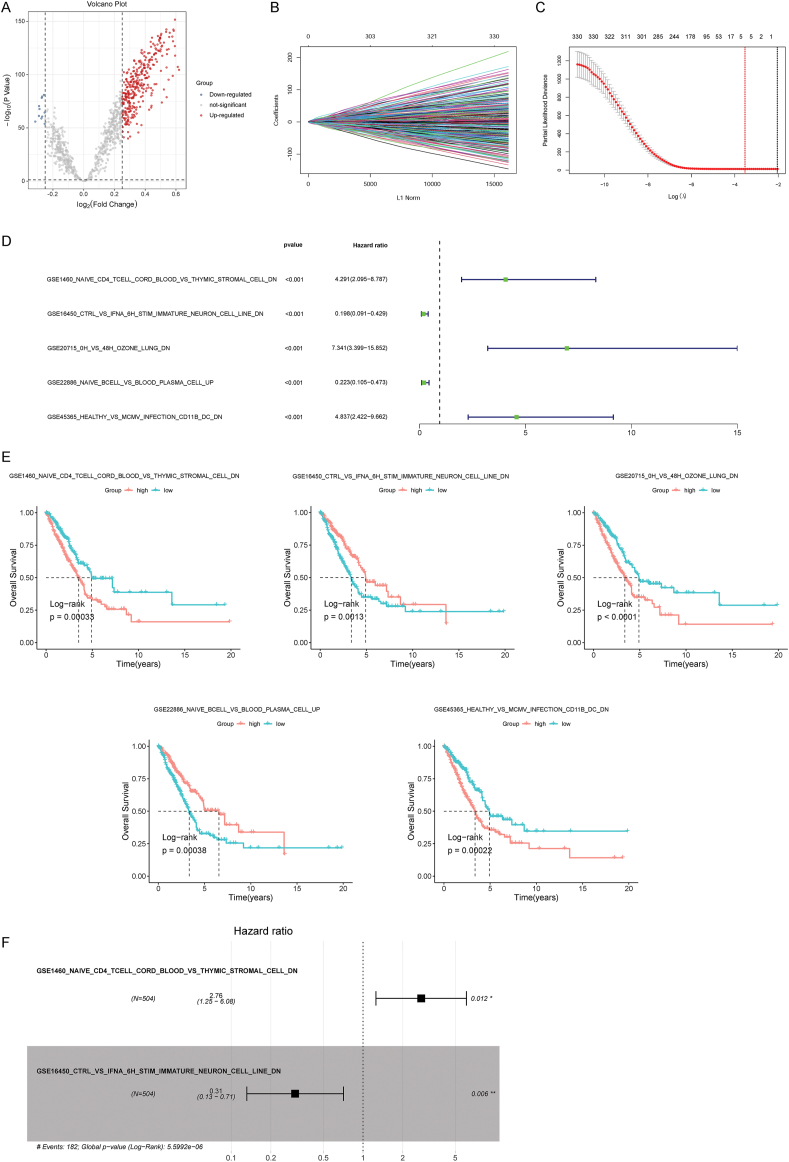
Identification of subtype-related differentially expressed genes (DEGs) and construction of a prognostic risk score (RS) model. A: Identification of subtype-related DEGs. B: Least absolute shrinkage and selection operator (LASSO) coefficient distribution. C: Likelihood deviation of LASSO coefficient distribution and the two dotted vertical lines represent lambda.min (left, red line) and lambda.1se (right, black line). D: Forest plots of univariate COX regression analysis of five gene sets. E: Kaplan–Meier (KM) survival analyses of the association of enrichment scores of five gene sets and OS. F: Forest plots of multivariate COX regression analysis of two gene sets in the prognostic RS model.

Based on the tumor subtype information, 331 DEGs between clusters 1 and 2 were identified (Fig. 5A). For subtype-related DEGs, LASSO regression analysis was performed, and five feature gene sets were obtained (Fig. 5B and C). Survival analysis further revealed that the high enrichment scores of GSE1460_NAIVE_CD4_TCELL_CORD_BLOOD_VS_THYMIC_STROMAL_CELL_DN, GSE20715_0H_VS_48H_OZONE_LUNG_DN, and GSE45365_HEALTHY_VS_MCMV_INFECTION_CD11B_DC_DN were associated with poor OS, whereas the high enrichment scores of GSE16450_CTRL_VS_IFNA_6H_STIM_IMMATURE_NEURON_CELL_LINE_DN and GSE22886_NAIVE_BCELL_VS_BLOOD_PLASMA_CELL_UP were related to better OS (Fig. 5D and E). Next, stepwise Cox regression analysis identified two gene sets, GSE1460_NAIVE_CD4_TCELL_CORD_BLOOD_VS_THYMIC_STROMAL_CELL_DN and GSE16450_CTRL_VS_IFNA_6H_STIM_IMMATURE_NEURON_CELL_LINE_DN (Fig. 5F). Based on the regression coefficient and enrichment scores in TCGA-LUAD dataset, a prognostic RS model was developed.

### 3.5 The prognostic signature had good accuracy in predicting OS

**Fig. 6:**
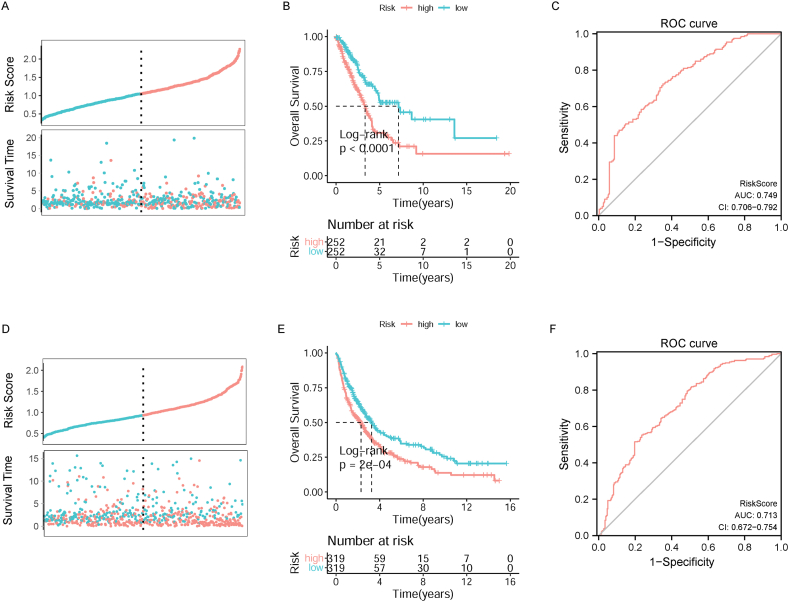
The prognostic RS model has good efficiency in predicting prognosis. A: RSs were calculated in samples in TCGA-LUAD dataset. B: KM survival analysis of high-risk and low-risk groups based on TCGA-LUAD dataset. C: Reactive oxygen species (ROC) analysis of the value of RS in predicting the survival of patients in TCGA-LUAD dataset. D: RSs were calculated in samples in GEO dataset. E: KM survival analysis of high-risk and low-risk groups based on GEO dataset. F: ROC analysis of the value of RS in predicting the survival of patients in GEO dataset.

The RS of each case in TCGA-LUAD training dataset was studied, and cases were stratified into high- and low-risk groups. Compared to lung cancer cases in the low-risk group, the OS in the high-risk group is shorter (Fig. 6A and B). The area under the receiver operating characteristic (ROC) curve (AUC) value reached 0.749 (95% CI: 0.706–0.792), suggesting that the RS had a fine prediction effect for OS (Fig. 6C). Besides, the GEO validation dataset was performed to test the robustness of the RS model. Likewise, the survival time of cases was obviously reduced in the high-risk group (Fig. 6D and E). The AUC value of the RS was 0.713 (95% CI: 0.672–0.754) (Fig. 6F).

### 3.6 A nomogram was constructed via the RS and clinicopathology factors

**Fig. 7:**
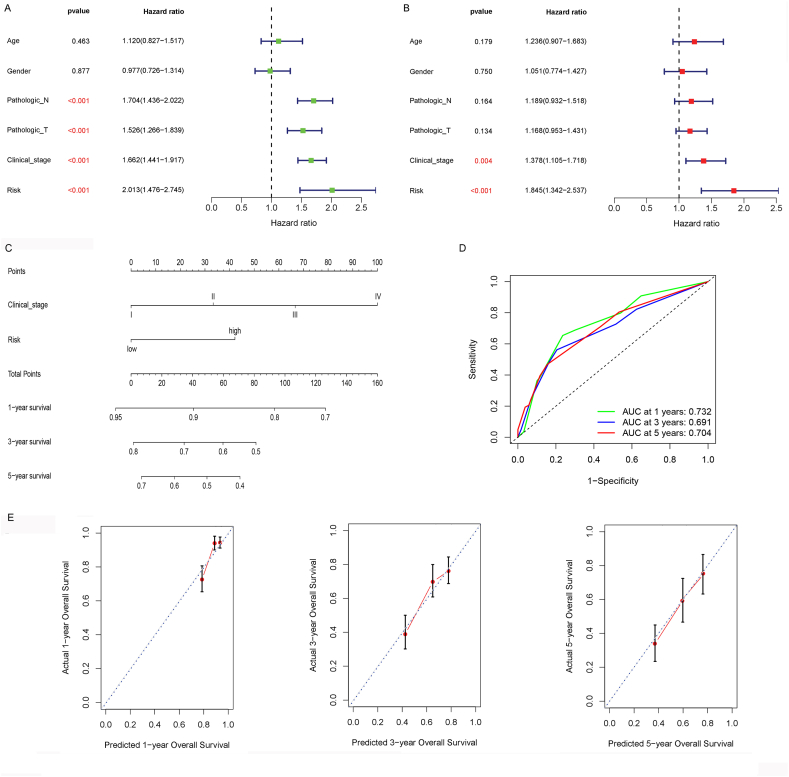
The RS was an independent prognostic factor and nomogram was constructed based on the RSs and clinical factors. A: Forest plots of univariate Cox regression analysis. B: Forest plots of multivariate Cox regression analysis. C: The constructed nomogram can predict the survival of patients with lung cancer. D, ROC curve for the predicting 1-, 3-, and 5-year survival probabilities. E: Calibration curve of nomogram for predicting the 1-, 3-, and 5-year survival probabilities. X-axis represents the nomogram-predicted probability and y-axis represents the actual survival.

The independent prognostic factors were further investigated. Univariate Cox regression analysis revealed that the RS, pathologic N, pathologic T, and clinical stages were obviously related to OS (*P* < 0.001, Fig. 7A). Next, multivariate Cox regression analysis revealed the RS (*P* = 0.004) and clinical stage (*P* < 0.001) as independent prognostic marker for cases with lung cancer (Fig. 7B). Then, a nomogram was established, which incorporated the RS and clinical stage to predict the 1-, 3-, and 5-year survival probabilities (Fig. 7C). C-index of this nomogram was 0.71 (95% CI = 0.661–0.758). AUC of the nomogram model for predicting the 1-, 3-, 5-year survival rate was 0.732, 0.691 and 0.704, respectively (Fig. 6D). Calibration curve analysis confirmed the good predictive value of the nomogram (Fig. 7E).

**Fig. 8:**
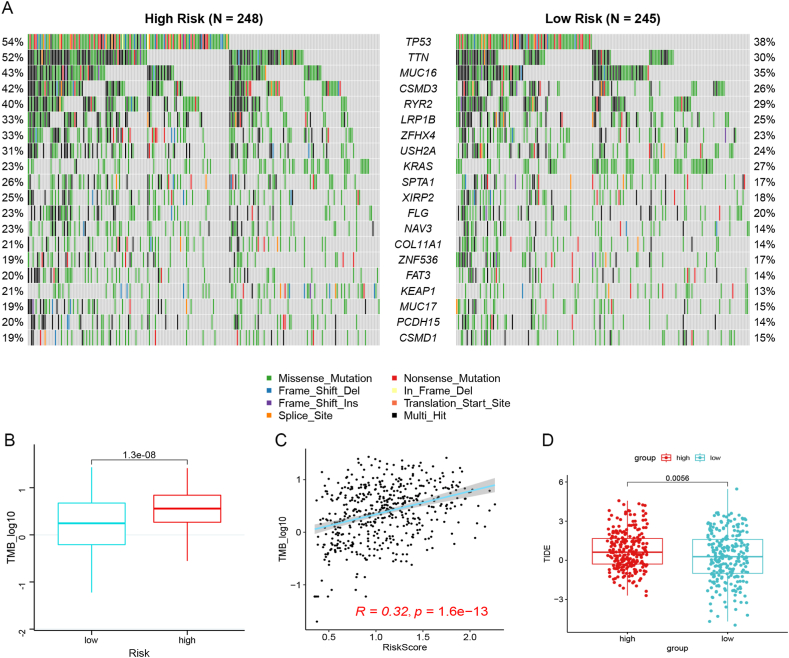
The RS was positively correlated with the tumor mutation burden (TMB). A: Comparison of the mutational frequency of top 20 mutated genes between high and low risk groups. B: Comparison of TMB levels of high- and low-risk groups. C: Correlation analysis of TMB levels with RS. D, Comparison of TIDE between high and low risk group.

*Difference of mutation status and immunotherapy response between high and low risk group* Based on the mutation information of samples in TCGA-LUAD dataset, the mutation frequencies of all the genes were obtained. As shown in Fig. 8A, the genes with the top 20 mutation frequencies are listed, all of which represent higher mutation frequencies in high-risk group than in low risk group, such as *TTN* and *TP53*. Moreover, the TMB of all tumor samples was calculated, and the TMB in the high-risk samples was obviously higher than that in the low-risk specimens (p < 0.05, Fig. 8B). Relationship analysis indicated that the TMB levels in specimens were obviously related to the RS (*P* = 1.6e-13, Fig. 8C). For immune response evaluation, TIDE score was calculated. As depicted in Fig. 8D, TIDE was obviously higher in high risk group than that in low risk group (*P* = 0.0056), indicating the low response to immunotherapy in cases with high risk.

## 4 Discussion

LUAD is a complex disease, therefore, different cases may present different treatment outcomes at the same pathological stage; some can overcome the disease, while others may show a relapse. Treatment regimens targeting anaplastic lymphoma kinase and epidermal growth factor receptor have benefited only a small percentage of cases with LUAD over the past decade [26,27]. Identifying the molecular subtypes of LUAD is urgently needed for its precise treatment [28]. In this study, we uncovered two molecular subtypes of LUAD according to the activity changes of the hallmark and immunologic gene sets in LUAD specimens from TCGA-LUAD cohort. Cluster 2 had worse prognosis, more advanced TN and clinical stages, and higher immune infiltration than cluster 1. Moreover, we established a prognostic signature using two LUAD subtype-related DGSs, which could stratify patients into different risk groups, and cases in the high-risk group had a reduced survival time and high TMB levels.

Several studies have focused on the exploration of LUAD subtypes due to the significant heterogeneity of this cancer type. For example, Xu et al. identified three immune subtypes of LUAD on the basis of the levels profiling of 29 immune-related gene sets, which provide potential components for LUAD treatment by targeting the immune signature [29]. Wang et al. determined two distinct subtypes of LUAD based on 433 immune-related genes, which provided implications for immune checkpoint blockade therapy [30]. However, the prognostic value of immune related genes and hallmark genes for LUADs is less known. Consistent with the previous report [30], we identified two LUAD subtypes with obviously different prognostic implication on the basis of the activity changes of the hallmark and immunologic gene sets. Furthermore, RS model was constructed in our study based on DEGs between subtypes. Our RS model presented promising prediction effect of the prognosis of LUAD. Different from the previous studies, we identified that RS was an independent risk factor for prognosis, and the RS model might benefit discriminate cases with high risk and low risk, guiding the personalized treatment.

Our data showed that worse OS was found in patients with high-RSs, suggesting that more regular follow-up examinations are required to monitor the disease occurrence in these patients. In addition, the RS could detached predict OS in cases with LUAD, hinting at its significant value in clinical decision making. Moreover, a nomogram can estimate the survival of cases with cancer [28]. For a more intuitive clinical application, we structured a nomogram that incorporated the RSs and clinicopathology features, including clinical stage, which exhibited good accuracy in predicting prognosis. This nomogram provides potent evidence for prognostic value of RS model in predicting survival and its utilization in guiding individualized treatment strategies for patients.

Furthermore, our constructed prognostic signature could divide patients into high and low risk groups, and high TMB levels were observed in the high-risk patients. TMB is an indicator for predicting responses to immune checkpoint blockade (ICB) treatment [31,32]. An comprehensive analysis of 27 types of cancer shows a obviously correlation between TMB levels and ICB treatment [33]. Three previous clinicopathology trials, including CHECKMATE-026, KEYNOTE-001, and CHECKMATE-227, revealed that cases with NSCLC with high TMB levels could benefit more from ICB treatment [[34], [35], [36]]. In current clinical practice, few patients are found to benefit from ICB therapy; therefore, it is urgent to discover a novel approach to screen patients sensitive to ICB treatment. Our results revealed higher TMB levels in high-risk cases, implying that these cases might be more sensitive to ICB therapy. In addition, *TP*53 and *TTN* were found to be frequently mutated in LUAD samples, and their mutation frequencies were higher in the high-risk samples than the low-risk samples. *TP53* mutation has been revealed to be a bad prognostic marker in cases with LUAD [37]. Moreover, *TP53-*mutated tumors exhibit remarkably increased TMB in LUAD, suggesting that LUAD patients with *TP53* mutations might derive clinical benefits from ICB therapy [38]. According to reports, *TTN* mutations are common in many types of cancer and are related to TMB status [39,40]. Xue et al. suggested that TTN/TP53 mutations could function as predictors of chemotherapy response in LUAD [41]. Thus, we speculate that *TTN/TP53* mutations may be responsible for the reaction to ICB therapy in cases with LUAD with high-RSs.

Our research has several deficiencies. First, our data was concluded using online public data, and the robustness of our constructed prognostic signature warrants further verification in more cohorts. Second, the clinical application of our identified prognostic RS model was not validated in clinical samples. Thus, a large number of LUAD samples are required and sequenced to determine whether RS model exerts strong capacity to discriminate LUAD with high risk from those with low risk. In conclusion, two LUAD subtypes were identified based on their prognostic hallmarks and immunologic gene sets. The prognostic markers we constructed could exactly predict the prognosis and immune microenvironment of cases with LUAD. Furthermore, the RS model may facilitate prognosis prediction and improve personalized therapeutic strategies for cases with LUAD.

## Ethics approval and consent to participate

TCGA is a public databases. The cases concerned with the database have acquired ethical approval. Scholars can download relevant data for free for research and publish relevant articles. Our research is based on open source data, so there are no ethical issues and other conflicts of interest.

## Consent for publication

Not Applicable.

## Availability of data and materials

Previously reported gene expression and clinical data were performed to support this research and are available at the Cancer Genome Atlas (TCGA, https://portal.gdc.cancer.gov/). These prior studies (and datasets) are cited at relevant places within the text as references.

## Funding

Not Applicable.

## CRediT authorship contribution statement

**Shun-Kai Zhou:** Conceptualization, Data curation, Writing – original draft. **De-Hua Zeng:** Formal analysis, Methodology, Writing – original draft. **Mei-Qing Zhang:** Data curation, Investigation, Writing – review & editing. **Meng-Meng Chen:** Investigation, Software, Writing – review & editing. **Ya-Ming Liu:** Methodology, Resources. **Qi-Qiang Chen:** Investigation, Validation. **Zhen-Ya Lin:** Project administration, Visualization. **Sheng-Sheng Yang:** Software, Supervision. **Zhi-Chao Fu:** Resources, Software, Visualization. **Duo-Huang Lian:** Project administration, Visualization. **Wen-Min Ying:** Conceptualization, Project administration, Supervision.

## Declaration of competing interest

The authors declare that they have no known competing financial interests or personal relationships that could have appeared to influence the work reported in this paper.
